# Prenatal ultrasound manifestations and classification of 37 fetuses with limb–body wall complex: a retrospective study

**DOI:** 10.3389/fmed.2026.1731562

**Published:** 2026-02-02

**Authors:** Xining Wu, Kun Li, Ruijie Wang, Peipei Zhang, Yunshu Ouyang, Qing Dai, Yixiu Zhang, Hua Meng

**Affiliations:** 1 Department of Ultrasound, State Key Laboratory of Complex Severe and Rare Diseases, Peking Union Medical College Hospital, Chinese Academy of Medical Sciences and Peking Union Medical College, Beijing, China; 2 Department of Ultrasound, The Affiliated Jiangning Hospital of Nanjing Medical University, Nanjing, China; 3 Department of Ultrasound, Xuzhou Maternity and Child Health Care Hospital, Xuzhou Medical University Affiliated Maternity and Child Health Care Hospital, Xuzhou, China

**Keywords:** diagnosis, fetus, limb–body wall complex, prenatal, ultrasound

## Abstract

**Objective:**

This study aimed to provide a clinical reference for prenatal diagnosis by summarizing the ultrasound manifestations and classifications of fetal limb–body wall complex (LBWC).

**Methods:**

We retrospectively reviewed cases of LBWC diagnosed through prenatal ultrasound examination at Peking Union Medical College Hospital and Xuzhou Maternal and Child Health Hospital between 2012 and 2023. The primary prenatal ultrasound imaging features and associated malformations were recorded and classified into two categories based on the presence (type I) or absence (type II) of craniofacial anomalies.

**Results:**

Among 37 fetuses with LBWC, 4 were classified as type I, 31 were classified as type II, and 2 exhibited features of both type I and type II concurrently. All fetuses had varying degrees of thoracoschisis or gastroschisis with visceral herniation. A total of 35 fetuses had limb abnormalities, and 11 had craniofacial abnormalities. All fetuses showed varying degrees of spinal curvature, and 23 had umbilical cord abnormalities. In addition, 32 fetuses had other abnormalities, including a persistent extraembryonic coelom in 12 fetuses, an amniotic band in 9 fetuses, nuchal translucency thickening in 5 fetuses, nuchal cystic hygroma in 3 fetuses, an invisible bladder in 2 fetuses, and external genital anomalies in 1 fetus. All cases resulted in induced termination.

**Conclusion:**

Fetal LBWC has characteristic ultrasonographic features and can be diagnosed in the first trimester. An accurate prenatal ultrasound assessment is essential to enable clinicians to offer future parents the necessary information and counseling concerning the prognosis of this type of anomaly.

## Introduction

Limb–body wall complex (LBWC), also known as body stalk anomaly, is a severe fetal multi-malformation syndrome characterized by a major thoracoschisis or gastroschisis, visceral herniation, severe scoliosis or kyphoscoliosis, limb deformities, craniofacial malformations, and umbilical cord abnormalities (1). As LBWC is a lethal anomaly, affected pregnancies are often terminated (2).

The reported prevalence of LBWC varies widely, with estimates ranging from 0.04 to 3.3 per 10,000 births (3–6). This variability is attributed to factors such as differing diagnostic criteria, early spontaneous abortions, pregnancy termination after diagnosis, and advances in prenatal diagnostic technologies. With the rapid development of prenatal ultrasound technology, standardized ultrasound examinations in early pregnancy (11–13^+6^ weeks) have been widely promoted and applied. Thus, an increasing number of LBWC cases are definitively diagnosed in early pregnancy, providing parents more time to consider and determine pregnancy outcomes. However, there is limited literature discussing the prenatal ultrasound diagnosis of LBWC. This study retrospectively summarizes the ultrasound imaging features of fetal LBWC cases, explores the pathogenesis of LBWC, and aims to offer comprehensive guidance for the prenatal diagnosis of LBWC.

## Materials and methods

We retrospectively analyzed cases of LBWC diagnosed through prenatal ultrasound at Peking Union Medical College Hospital and Xuzhou Maternity and Child Health Care Hospital between 2012 and 2023. Using the hospitals’ database search function, we identified all cases with prenatal ultrasound findings suggestive of LBWC by applying the keywords “LBWC,” “body stalk anomaly,” or “amniotic band syndrome.” Two obstetric ultrasound experts with more than 15 years of work experience independently reviewed each case, combining prenatal ultrasound images, clinical medical records, and postnatal pathological reports to confirm the diagnosis of LBWC. Consensus was reached for all included cases. The institutional review board of Peking Union Medical College Hospital approved the study and waived the need for written consent from the patients. Xuzhou Maternal and Child Health Care Hospital handled the ethical filing.

Ultrasound examinations were performed using color Doppler ultrasound diagnostic instruments (GE Voluson E10/E8, GE Healthcare; and Philips EPIQ7/IU22, Philips Healthcare) equipped with a transabdominal two-dimensional or three-dimensional convex array probe with frequency ranges of 2.0–5.0 MHz and 1.0–5.0 MHz, respectively. A transvaginal ultrasound probe with a frequency range of 4.0–8.5 MHz was also used.

All fetal ultrasound examinations were performed by certified sonographers who had obtained qualifications from the regional maternal and child health administration and followed the guidelines recommended by the International Society of Ultrasound in Obstetrics and Gynecology (ISUOG). The examinations involved sequential observations of fetal cranial, facial, thoracoabdominal, spinal, limb, and cardiac development. When a suspicious thoracoabdominal wall defect was detected, particular attention was paid to detect any abnormal echogenic masses, which included an assessment of the mass location, size, morphology, content, and presence/absence of a membrane, and a thorough assessment of concomitant limb structural anomalies/position abnormalities, craniofacial deformities, and changes in spinal physiological curvature. The integrity of the amniotic sac and its relationship to the fetus were carefully inspected, along with assessments of the umbilical cord length, course, and placental insertion site, and the extraembryonic cavity. Certain cases required more detailed assessment using transvaginal ultrasound and three-dimensional ultrasound with surface rendering.

## Results

### Clinical data

**Figure 1:**
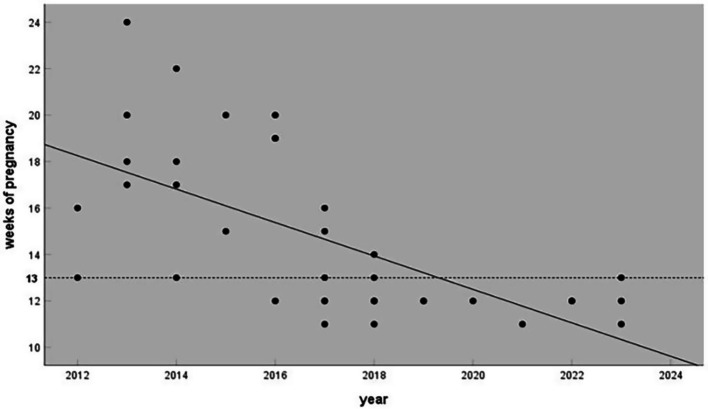
Gestational age of limb–body wall complex cases diagnosed at two hospitals from 2012 to 2023. Regression analysis revealed that the gestational age at diagnosis became earlier over time.

A total of 37 fetuses were diagnosed with LBWC during the study period. Among them, 33 fetuses were from singleton pregnancies, and 4 were from twin pregnancies. Two fetuses with LBWC were from twin gestations: one case of monochorionic–monoamniotic twins and one case of monochorionic–diamniotic twins. Additionally, there was one case of monochorionic–diamniotic twins in which both fetuses were affected by LBWC (this case was considered as two separate fetuses with LBWC). The mean maternal age was 28.6 ± 5.3 years (range 19–39 years). Figure 1 shows the gestational ages at which the fetuses were diagnosed with LBWC. The median gestational age at the initial detection of LBWC was 13 + 1 weeks (range 11 + 1 to 24 + 0 weeks). In 21 cases, LBWC was identified in early pregnancy ultrasound examinations conducted during 11 + 1 to 13 + 6 weeks of gestation. All cases had no abnormal family history. Genetic testing was performed in five cases, all of which showed normal fetal karyotypes. All patients opted to terminate their pregnancy (Table 1).

**Table 1:** Clinical data of 37 fetuses with limb–body wall complex.

Clinical data	*N* (%)
Maternal age (years, mean ± SD)	28.6 ± 5.3
From singleton pregnancies	33 (89.2%)
From twin pregnancies	4 (10.8%)
MCMA	1 (2.7%)
MCDA	3 (8.1%)
Gestational week at the time of diagnosis (median, range)	13^+1^ (11^+1^, 24^+0^)
Type	
Type I	4 (10.8%)
Type II	31 (83.8%)
Both Type I and Type II	2 (5.4%)
Genetic testing	5 (13.5%)
Termination of pregnancy	37 (100%)

MCMA, monochorionic–monoamniotic; MCDA, monochorionic–diamniotic.

### Fetal abnormalities

According to Russo et al. (7), LBWC is categorized into two types based on the presence or absence of craniofacial anomalies: Type I presents as craniofacial defects, usually associated with cranioplacental adhesions and/or amniotic bands. Type II does not exhibit craniofacial defects but shows urogenital anomalies, anal atresia, a short and fixed umbilical cord, and abdominal placental attachment, together with a persistence of the extraembryonic coelom. In the present case series, 4 cases were classified as type I (placental-cranial type), 31 were classified as type II (placental-abdominal type), and 2 were classified as features of both type I and type II concurrently. The ultrasound features associated with LBWC are outlined below and summarized in Table 2.

**Table 2:** Abnormal sonographic findings of the 37 fetuses.

	Thoracoschisis or gastroschisis with visceral herniation	Limb anomalies	Cranio-facial defects	Spinal anomalies	Umbilical cord anomalies	Other anomalies
Number (%)	37 (100%)	35 (94.6%)	11 (29.7%)	37 (100%)	23 (62.2%)	32 (86.5%)
Main sonographic findings	Significant thoracoabdominal wall defects; herniation of liver, stomach, and bowel loops	Partial or complete absence of limbs; clubfoot or positional limb deformities	Exencephaly or encephalocele; cleft lip and palate; invisible nose bone	Abnormal spinal curvatures; sacral agenesis; suspected spinal meningocele	Short rudimentary or no free umbilical cords; single umbilical artery	Persistent extra-embryonic coelomic cavity; amniotic band; nuchal translucency thickening; nuchal cystic hygroma; external genitalia anomalies; invisible bladder

#### Thoracoschisis or gastroschisis with visceral herniation

**Figure 2:**
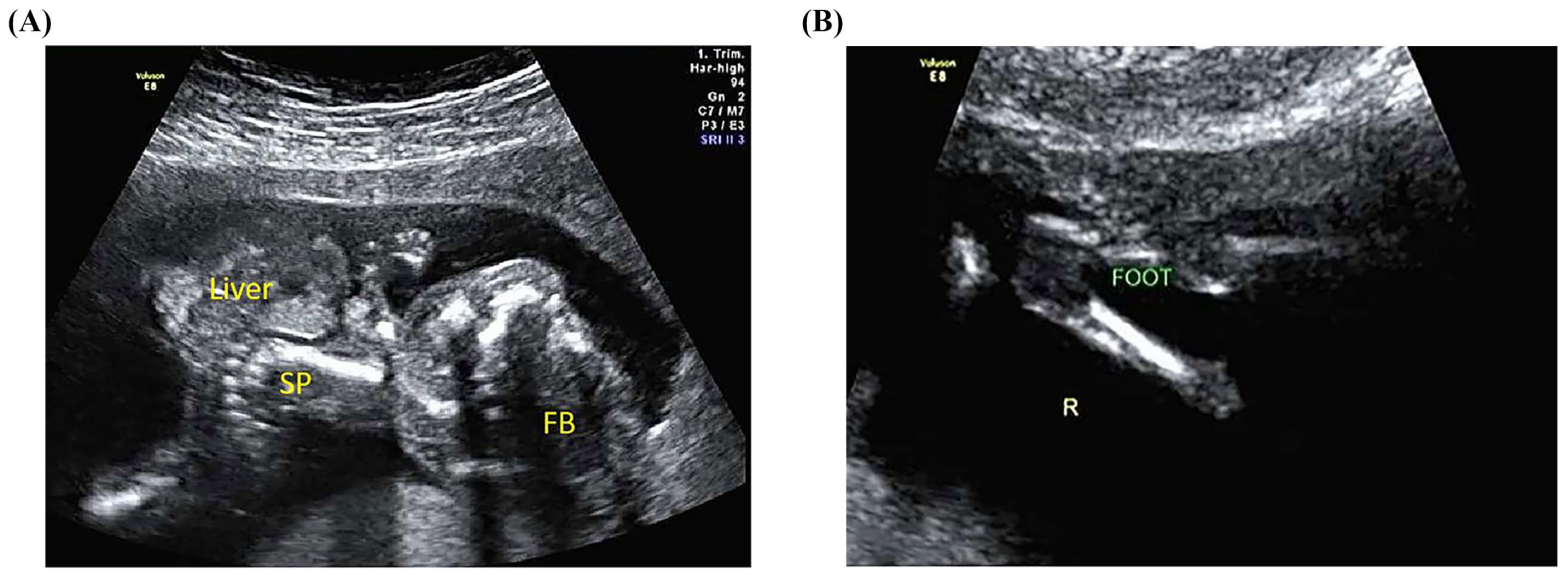
Ultrasound images of a fetus with limb–body wall complex in a 36-year-old woman. **(A)** The grayscale ultrasound shows the absence of the cranial bone halo, severe kyphoscoliosis of approximately 60 degrees, and herniation of abdominal viscera with an inner echo similar to the liver. SP: spine, FB: fetal brain. **(B)** Normal right foot morphology is not clearly visible on grayscale ultrasound. R: right.

All 37 fetuses presented with significant thoracoabdominal wall defects of varying severity. The viscera herniated through the defect included the liver (Figure 2), the stomach, and the bowel loops. The heart was partially extruded in six cases, while the kidneys were extracorporeal in two cases. The herniated viscera lacked a covering membrane, were located in the extraembryonic coelomic cavity, and were attached to the placenta or the uterine wall in four cases.

#### Limb anomalies

Thirty-five cases had limb anomalies. These anomalies predominantly affected the lower limbs, with only four cases exhibiting anomalies in the upper limbs. Twenty-one cases involved partial or complete absence of limbs, five had clubfoot and/or positional limb deformities, and nine presented with both limb absence and clubfoot or positional limb deformities. Additionally, eight cases of limb malrotation were discovered postnatally through pathological examination but were not detected on prenatal ultrasound.

#### Craniofacial defects

Eleven cases had craniofacial defects. Among these cases, eight exhibited loss or incompleteness of the fetal cranial halo, presenting as exencephaly or encephalocele (Figure 2). Inadequate visualization of a unilateral nasal bone was noted in one case, while a cleft lip and palate were suspected in another. Additionally, postnatal pathological examination revealed a midline cleft lip and palate in one case.

#### Spinal anomalies

Spinal anomalies of varying degrees of severity were present in all cases, encompassing two cases of sacral agenesis and 35 cases of abnormal spinal curvature, such as scoliosis or kyphoscoliosis (Figure 2). One case had an anechoic mass in the sacrococcygeal region, strongly suggesting spinal meningocele.

#### Umbilical cord anomalies

**Figure 3:**
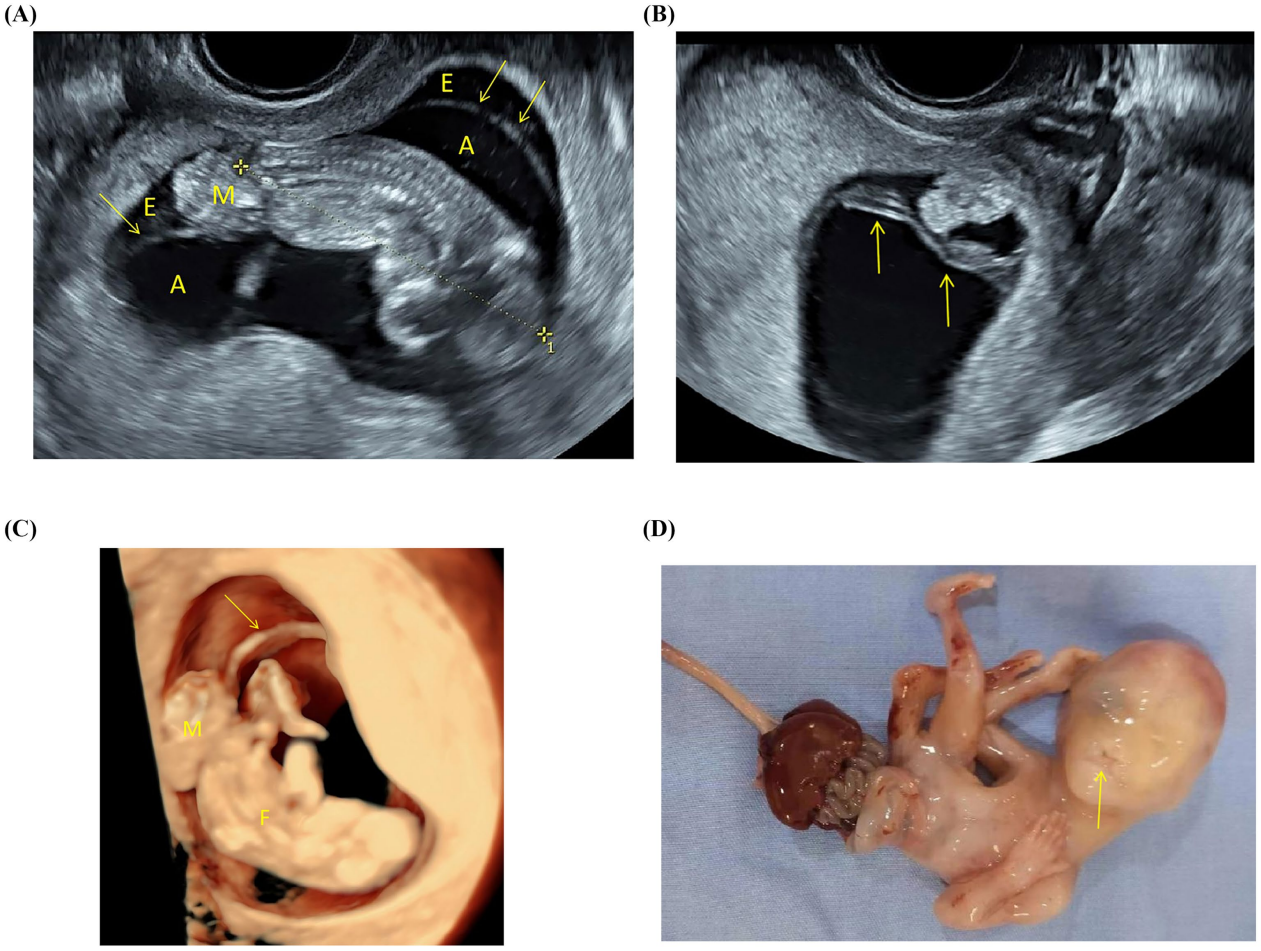
Routine fetal ultrasound examination of a 32-year-old woman in the first trimester. **(A)** Grayscale ultrasound shows that the fetal crown-rump length is shorter than expected for the gestational age in weeks. There is a large abdominal wall defect, with abdominal contents herniating into the unobliterated extraembryonic coelom (the arrows show the amniotic sac). A-amniotic cavity; E-extraembryonic coelom; M-mass. **(B)** The umbilical cord is short (arrows), lacks coiling, and is closely adhered to the amniotic membrane. **(C)** Three-dimensional ultrasound shows an abdominal extrusion of the fetus within the extraembryonic coelom (the arrow shows the amniotic sac), along with one lower limb displaying an abnormal posture. F-fetus; M-mass. **(D)** Gross examination follows induced labor demonstrates a fetal abdominal wall defect with visceral protrusion, limb malrotation, and a very short umbilical cord. Additionally, the fetus has a midline cleft lip and palate (arrow).

Twenty-three cases had fetal umbilical cord abnormalities. Among these cases, 19 exhibited a short, rudimentary umbilical cord (Figure 3), absence of coiling, and a lack of free umbilical cord, with the umbilical vessels running within the extraembryonic coelom. In the remaining 4 cases, a single umbilical artery was identified.

#### Other anomalies

A persistent extraembryonic coelomic cavity with a protruding mass located within it was observed in 12 cases (Figure 3). An echogenic band in the amniotic cavity was observed in nine cases, with nuchal translucency thickening (4.5–6.3 mm) observed in five cases. Three cases had nuchal cystic hygroma, two cases had an invisible bladder, and one case had external genital anomalies.

## Discussion

LBWC is a rare and severe malformation syndrome. The diagnostic criteria for LBWC vary across different reports (3, 8), leading to a confusing array of terms used to describe this condition (2). In the present study, we adhered to the definition proposed by Van Allen et al. (9), in which the diagnosis of LBWC is based on the presence of two out of three characteristic defects: thoracoschisis or gastroschisis with a large visceral herniation, limb defects, and exencephaly/encephalocele with facial clefts.

The etiology of LBWC remains elusive, and several competing pathophysiological theories have been proposed (6, 10, 11). In 1930, Streeter (12) proposed the intrinsic abnormal embryonic development theory, which suggests that LBWC results from a completely faulty trilaminar embryonic disc folding process along all three axes (cephalic, caudal, and lateral) during the first 4 weeks of embryonic development; this abnormal longitudinal folding results in thoracoschisis or gastroschisis. In 1965, Torpin (13) proposed the extrinsic theory, which suggests that early amniotic membrane rupture caused by direct mechanical pressure creates constriction bands that entangle the embryo, leading to severe deformities such as craniofacial defects, amputations, and body wall defects. In 1987, Van Allen et al. (9) introduced the early vascular disruption theory, which posits that a vascular disruption event occurring between the fourth and sixth weeks of gestation interrupts the normal blood supply to the developing embryo, leading to a wide range of anomalies affecting various organ systems. LBWC may also have a heterogeneous etiology, with several or all pathogenetic mechanisms being responsible in a subset of patients (14–17). In the present study, a midline cleft lip was found postnatally in one case (Figure 3d), without any associated membranous structure, thereby supporting the theory of an intrinsic cause. This finding also suggests that LBWC cannot be explained by a single mechanism and that different pathological mechanisms may arise from different phenotypes, warranting further research.

Due to the variability in clinical presentations of LBWC, different classifications have been proposed (7, 18, 19). Russo et al. (7) suggested dividing LBWC into two main phenotypes. Type I is characterized by craniofacial defects (exencephaly or cephalocele), facial clefts, cranial placental attachment, thoracoschisis, and upper limb abnormalities. Type II is characterized by thoraco-gastroschisis or gastroschisis, placento-abdominal attachments, lower limb abnormalities, urogenital anomalies, anal atresia, lumbosacral meningocele, scoliosis, and a short umbilical cord. In the present study, the higher prevalence of type II cases may reflect the greater severity of type I cases, which involve craniofacial abnormalities and often lead to early miscarriage. However, two cases in our series could not be clearly classified into either category. Sahinoglu et al. (16) have suggested that all previously proposed causative mechanisms may contribute, leading to different subgroups. They proposed a new phenotypic classification dividing LBWC into three types: type 1, a fetus with a craniofacial defect and an intact thoracoabdominal wall; type 2, a fetus with a supraumbilical, large thoracoabdominal wall defect; and type 3, a fetus with an infraumbilical abdominal wall defect with an intact thorax. Nevertheless, the majority of the proposed classifications are based on clinical interpretations of examined cases that form phenotype patterns, making it challenging to definitively classify all cases (14).

LBWC encompasses a spectrum of diseases with diverse phenotypes and varying degrees of severity. All of our cases exhibited extensive thoracoabdominal defects with visceral herniation, which are the most common and severe malformations in LBWC and the main abnormalities observed during ultrasound examinations (20). Limb anomalies have been accepted as a diagnostic criterion for LBWC (8). The rarity of upper limb anomalies in our case series can be attributed to the lower frequency of type I LBWC (21). Limb anomalies encompass a range of structural abnormalities, such as amelia, amputation, hypoplasia of all or part of a limb, and phocomelia of the pelvic limbs, and non-structural abnormalities, including simple clubfoot, arthrogryposis, ankyloses, and limb malrotation. Some authors propose that only severe limb reduction defects (amelia) should be considered as a diagnostic criterion (18, 22). However, other authors have also included minor defects, such as clubfoot, polydactyly, and oligodactyly (8, 23). As the third diagnostic key point, the cranial abnormalities observed in the present study predominantly encompassed exencephaly and encephalocele, both of which are lethal conditions that were detected during early or middle pregnancy. All 37 fetuses with LBWC in the present study displayed spinal developmental abnormalities. It is believed that aplasia or hypoplasia of the paraspinous or thoracolumbar musculature may contribute to the development of scoliosis (17). Extrusion of intra-abdominal contents and adhesion of the fetal abdomen to the placenta may result in restricted movement, causing the fetus to be in a flexed or hyperextended posture, leading to the asymmetric development of the spine.

The fetal appendages should also be carefully observed. The present study involved 23 cases with umbilical cord abnormalities, primarily characterized by a short (<10 cm) umbilical cord and a direct connection of the abdominal protrusion to the placenta (17). Umbilical cord abnormalities causing issues with the fetal blood supply may also contribute to embryonic fusion disorders. The presence of a short or absent umbilical cord and spinal abnormalities may serve as important radiological signs for diagnosing LBWC (1). Another characteristic feature of LBWC is the persistent presence of the extraembryonic coelom, manifested as a large cystic structure or fetal abdominal protrusion at the site of the abdominal wall defect, with the lower half of the body located outside the amniotic cavity. This is due to the failure of the embryo to fold along the craniocaudal and lateral axes to form a cylindrical shape (2).

The diagnosis of LBWC can typically be confirmed via prenatal ultrasound by the end of the first trimester (1, 17, 24). It is essential to conduct a comprehensive anatomical examination of the fetus while measuring nuchal translucency (25, 26). During early pregnancy, the extraembryonic coelom is relatively large, providing a clear view of intra-abdominal organs protruding into the cavity and the abdominal–placental attachment (5). Severe scoliosis can also be readily identified during the first trimester (1, 27). LBWC is accepted as a fatal anomaly and often results in intrauterine fetal demise or early neonatal death (28), making it crucial to distinguish LBWC from other potentially treatable anterior wall defects (2). Gastroschisis is characterized by a small paraumbilical defect on the right side, with the abdominal organs everted into the amniotic cavity. An omphalocele involves a supraumbilical defect with the intestinal loops and liver protruding into the umbilical cord, covered by the peritoneum and amniotic membrane. In bladder and the cloacal exstrophy, the bladder is not visible, and the bowel loops protrude between the two halves of the bladder (4). Fetuses with these malformations typically have normal umbilical cord length and usually do not have spinal and limb developmental abnormalities. However, it is challenging to differentiate between LBWC and amniotic band syndrome (ABS) due to the lack of clear diagnostic criteria (3, 14, 15). ABS is known to occur as a consequence of early amniotic membrane rupture and can present as a wide range of defects, such as craniofacial and limb malformations. Some researchers suggest that ABS and LBWC may belong to the same phenotypic spectrum with overlapping features, while others propose that LBWC is a distinct entity with the amniotic bands produced earlier (15, 29).

This study reviewed cases of LBWC in the past 12 years. Although the sample size was not large and some data were incomplete, the findings contribute to understanding the ultrasound manifestations of LBWC and to expanding the phenotypic spectrum of LBWC. Moreover, since 2018, LBWC has been increasingly detected and diagnosed before the 14th week, a trend attributed to the popularization of nuchal translucency examination. This finding also reflects the progress of ultrasound examination technology in the first trimester of pregnancy and its value in screening serious fetal structural malformations.

## Conclusion

LBWC has characteristic ultrasonographic features and can be diagnosed in the first trimester. Accurate prenatal ultrasound assessment is essential to provide clinicians with information needed to counsel future parents about the prognosis of this anomaly.

## Data Availability

The original contributions presented in the study are included in the article/supplementary material, further inquiries can be directed to the corresponding authors.
